# Clinically applicable artificial intelligence system for dental diagnosis with CBCT

**DOI:** 10.1038/s41598-021-94093-9

**Published:** 2021-07-22

**Authors:** Matvey Ezhov, Maxim Gusarev, Maria Golitsyna, Julian M. Yates, Evgeny Kushnerev, Dania Tamimi, Secil Aksoy, Eugene Shumilov, Alex Sanders, Kaan Orhan

**Affiliations:** 1 Diagnocat Inc, San Francisco, CA USA; 2 Division of Dentistry, School of Medical Sciences, The University of Manchester, Manchester, UK; 3 Private Practice, Orlando, FL USA; 4 Department of DentoMaxillofacial Radiology, Faculty of Dentistry, Near East University, Nicosia, Cyprus; 5 Department of DentoMaxillofacial Radiology, Faculty of Dentistry, Ankara University, 06500 Ankara, Turkey; 6 Medical Design Application and Research Center (MEDITAM), Ankara University, Ankara, Turkey

**Keywords:** Medical research, Dental diseases

## Abstract

In this study, a novel AI system based on deep learning methods was evaluated to determine its real-time performance of CBCT imaging diagnosis of anatomical landmarks, pathologies, clinical effectiveness, and safety when used by dentists in a clinical setting. The system consists of 5 modules: ROI-localization-module (segmentation of teeth and jaws), tooth-localization and numeration-module, periodontitis-module, caries-localization-module, and periapical-lesion-localization-module. These modules use CNN based on state-of-the-art architectures. In total, 1346 CBCT scans were used to train the modules. After annotation and model development, the AI system was tested for diagnostic capabilities of the Diagnocat AI system. 24 dentists participated in the clinical evaluation of the system. 30 CBCT scans were examined by two groups of dentists, where one group was aided by Diagnocat and the other was unaided. The results for the overall sensitivity and specificity for aided and unaided groups were calculated as an aggregate of all conditions. The sensitivity values for aided and unaided groups were 0.8537 and 0.7672 while specificity was 0.9672 and 0.9616 respectively. There was a statistically significant difference between the groups (p = 0.032). This study showed that the proposed AI system significantly improved the diagnostic capabilities of dentists.

## Introduction

Radiological examination is an essential part of patient management in dentistry. It is frequently used to supplement and aid clinical diagnosis of pathology related to teeth and adjacent structures^1–4^. Cone-beam computed tomography (CBCT) was proposed for maxillofacial imaging^5,6^ during the last decade and is now becoming increasingly popular for such use. It offers distinct advantages including lower radiation doses, compared to medical computed tomography (CT), and the potential of importing and exporting individualized, digital imaging and communications in medicine (DICOM) and overlap-free reconstructed data for other applications^4–7^. CBCT can supply high-resolution three-dimensional (3D) images without distortion and superimposition of bone and other dental structures that can be seen in conventional radiography^8,9^.

Several studies have compared the diagnostic accuracy of CBCT with conventional and digital panoramic and periapical radiography^10–14^. CBCT has been shown to significantly increase the detection rate of tooth root canal spaces and periapical areas for the evaluation of dental infection and pathology compared to conventional imaging^15–17^. This suggests that CBCT enhances the recognition of periapical bone lesions and offers improved diagnostic accuracy, treatment planning, and thus prognostic outcomes. These and other possibilities along with increasing access to CBCT imaging for dentists are allowing the transition from 2 to 3D imaging in everyday dental practice^10–17^.

Previous studies revealed that CBCT has a wide application in the field of dentistry^18–20^. However, there is no standard curriculum or protocol for providing training regarding CBCT. The current status of awareness and knowledge concerning CBCT amongst dental practitioners is not known precisely^21^.

There have been limited works in the literature studying the knowledge and attitude of dentists toward advanced dentomaxillofacial imaging. The literature showed that there is a lack of knowledge regarding CBCT^22,23^. It should also be pointed out that studying CBCT should take more time in dental school curricula^21^. Reddy et al.^23^ in their work defined very low awareness amongst the dentists regarding CBCT applications, which can be interpreted as a lack of experience in that area. Thus, computer-aided systems have been developed to assist in medical and dental imaging diagnosis^24–27^.

Along with the expansion of CBCT, radiation-related effects of CBCT imaging raise concerns about its use in dentistry. CBCT is associated with a higher radiation dose compared to panoramic and intraoral imaging, but a lower dose compared to conventional tomography^28–30^. Therefore radiation risk assessment is in place. The effective dose recommended by ICRP (International Commission on Radiological Protection) should be kept accordingly to the principles of ALARA (As Low as Reasonably Achievable) and ALADA (As Low as Diagnostically Acceptable). It should also be stated that the necessity of CBCT scanning must be indication-oriented and patient specific^29^.

Convolutional Neural Networks (CNN) are most commonly used for object detection and segmentation. There are several studies available with deep-learning methods, including CNNs, to assist clinicians in dentistry. CNNs are used clinically for, apical lesion detection^31^, detection of root fractures^32^, detection of periodontal disease^33^, cystic lesions^34^, caries detection^35^, staging of lower third molar development^36^, tooth detection^37,38^, diagnosis of jaw lesion^39^, and other pathologies detection^40^. Artificial intelligence (AI) provides an added value and decision support tool for such medical imaging.

There are a few critical success factors to measure the gap between actual performance and expected achievement. AI systems must apply to real-world situations and be designed for clinical evaluation and deployment. Furthermore, an important part of the development and integration of these AI systems is that their functionality (ease of use, speed, and accuracy) reaches or exceeds the clinicians' expertise and expectations.

In this study, a novel AI system, which is based on deep learning methods, was tested for diagnostic capabilities. Firstly, the clinical performance, accuracy, and time required for diagnosis were evaluated. The real-time performance of CBCT imaging was evaluated on the diagnosis of anatomical landmarks and pathologies. Secondly, its clinical effectiveness and safety were tested when used by dentists in a clinical setting. The null hypothesis of this study was that there is no significant difference between aided and unaided groups using the proposed AI system (Diagnocat) for CBCT imaging.

## Results

### Inter-observer consistency

Table 1 shows the ICCs between observers which ranged from 0.59 to 0.99. There was a high inter-observer agreement, while a high ICC and low CV demonstrated that the procedure was standardized between the evaluations and measurements of the observers. No statistical differences were found among observers' evaluations (p > 0.05) except for caries, periodontal ligament (PDL) widening along the root, and periodontal bone loss diagnosis (p < 0.05).

**Table 1:** Inter-observer agreement among observers according to evaluated variables.

Groups	Observers (mean)	
	ICC	CV (%)
Artificial crown	0.942	2.3
Canals N = 1	0.941	2.11
Canals N = 2	0.952	2.01
Canals N = 3	0.962	2.05
Canals N = 4	0.962	2.05
Canals N = 5	0.962	2.05
Caries signs	0.592*	7.02*
Crown defect over 50pct	0.99	2.04
Endodontically treated tooth	0.988	2.05
Filling	0.99	1.05
Impaction	0.952	2.07
Implant	0.999	2.05
Missed canal	0.925	2.01
Missing	0.999	2.05
Overfilling	0.98	2.02
PDL widening along the root	0.690*	6.04*
Periapical lesion	0.887	2.98
The periapical lesion, PDL widening	0.589	6.02
Periapical lesion, poorly circumscribed radiolucency	0.555	6.64
Periapical lesion, radiopacity	0.889	2.8
Periapical lesion, well-circumscribed radiolucency	0.85	2.08
Periodontal bone loss	0.629*	5.80*
Periodontal bone loss, mild	0.78	4.8
Periodontal bone loss, moderate	0.76	4.8
Periodontal bone loss, severe	0.777	3.8
Pontic	0.887	2.76
Post and core	0.999	2.05
Roots N = 1	0.999	2.05
Roots N = 2	0.999	2.05
Roots N = 3	0.999	2.05
Roots N = 4	0.962	2.05
Short filling	0.875	2.02
Voids present in the root filling	0.887	2.56

CV, coefficient of variation; ICC, intraclass correlation coefficient.

*Indicates significant difference p-value less than 0.05.

The results of the AI evaluation are shown in Tables 2 and 3. Table 2 shows the overall sensitivity and specificity for the system and dentomaxillofacial radiology examiners. Outcome counting for Table 4 was summarized over the case, tooth, and condition, whilst grouped by the participants. Both sensitivity and specificity were recorded as higher for human examiners. Overall sensitivity values for human examiners ranged between 0.9318 and 0.9438 while the value for this AI system was 0.9239. Overall specificity values for ground truth examiners were between 0.9899 and 0.9946 while the value for this AI system was 0.9899. Table 3 shows sensitivity and specificity values for the system given per condition. The results of specificity values were high, with the lowest being 0.94 when determining missing tooth. Sensitivity values were condition-dependent, with the lowest values being around 0.7 for some difficult or subjective conditions such as endodontic treatment (missed canal, short filling, voids in root filling), and signs of dental caries (complex to diagnose using the CBCT). Notably, Diagnocat struggled to detect very rare anatomical configurations of the tooth e.g., 5 canals or 4 roots. Finally, a rare subtype of the periapical lesion, periapical radiopacity, did not register in the dataset. Yet, this subtype currently is not claimed as a diagnostic capability of the Diagnocat system.

**Table 2:** Cross-condition sensitivity and specificity for the system and dentomaxillofacial radiology examiners.

Participant	Sensitivity	Specificity
Diagnocat	0.9239	0.9899
DMFR-1	0.9411	0.9939
DMFR-2	0.9438	0.9931
DMFR-3	0.9318	0.9913
DMFR-4	0.9337	0.9946

**Table 3:** Sensitivity and specificity by condition for the AI system (Diagnocat).

Condition	Sensitivity	Specificity
Artificial crown	0.9546	0.9963
Canals N = 1	0.9864	0.9661
Canals N = 2	0.7759	0.9927
Canals N = 3	0.9531	0.9647
Canals N = 4	0.654	0.9952
Canals N = 5	N/A	0.9998
Caries signs	0.7285	0.9953
Crown defect over 50%	0.8734	0.9975
Endodontically treated tooth	0.9676	0.9953
Filling	0.9721	0.9921
Impaction	0.9137	0.9995
Implant	0.9727	0.9997
Missed canal	0.6695	0.9974
Missing	0.9824	0.9405
Overfilling	0.7831	0.9973
PDL widening along the root	0.8794	0.9863
Periapical lesion	0.8383	0.9953
The periapical lesion, PDL widening	0.7587	0.9807
Periapical lesion, poorly circumscribed radiolucency	0.6957	0.9942
The periapical lesion, well-circumscribed radiolucency	0.729	0.9974
Periodontal bone loss	0.9489	0.9661
Periodontal bone loss, mild	0.9321	0.9742
Periodontal bone loss, moderate	0.9111	0.9866
Periodontal bone loss, severe	0.9286	0.996
Pontic	0.9101	0.9998
Post and core	0.75	0.9975
Roots N = 1	0.9593	0.9888
Roots N = 2	0.9181	0.9778
Roots N = 3	0.964	0.9786
Roots N = 4	0.0	1.0
Short filling	0.6981	0.9937
Voids present in the root filling	0.7329	0.9957

**Table 4:** Condition descriptions.

Condition	Description
Artificial crown	A tooth was restored with an artificial crown
Number of canals	Number of root canals in a tooth (1–5)
Caries signs	Signs of dental caries (cases where caries is certain, and there is a low chance of confusion with a metallic artifact or non-contrast filling)
Crown has defect over 50%	A crown is largely destroyed: at least 50% of the crown is missing
Endodontically treated tooth	A tooth displays signs of previous endodontic treatment
Filling	A crown was restored with a filling
Impaction	A tooth is impacted (unerupted)
Implant	There is an implant in place of a tooth
Missed canal	A root canal was missed (not filled) during endodontic treatment. Should be specified only if a tooth was endodontically treated
Missing	Absence of tooth, implant, and pontic under specified number including both extracted teeth and teeth that are not visible in the FoV of an image
Overfilling	Filling material is visualized beyond a radiographic apex. Should be specified only if a tooth was endodontically treated
Periapical lesion	Presence of inflammatory periapical lesion adjacent to one or more roots of a tooth
Pontic	There is a pontic restoration in place of a tooth (either base or middle part)
Post and core	A tooth was restored with a post and core restoration
Number of roots	Number of roots in the tooth (1–4)
Short filling	Root canal filling is short (ends in 2 mm or more from a radiographic apex). Should be specified only if a tooth was endodontically treated
Voids present in the root filling	A root canal contains voids (spaces that were not filled during previous endodontic treatment). Should be specified only if a tooth was endodontically treated

In Table 5 the results of the study are presented. Sensitivity and specificity values are shown for aided versus unaided reads for each condition. The lowest sensitivity values for the aided group were 0.1818 and 0.3535, detecting the roots (n = 4) and periodontal bone loss. The lowest specificity value was 0.8111 for periodontal bone loss. For the unaided group, the lowest sensitivity value was 0.2045 for periapical lesion and poorly defined radiolucency, the smallest specificity value was 0.7973 for caries. The highest sensitivity and specificity for both groups was 1 for implant detection. The results for the overall sensitivity and specificity for aided and unaided groups, calculated as an aggregate of all conditions. The sensitivity values for aided and unaided groups were 0.8537 and 0.7672 while specificity was 0.9672 and 0.9616 respectively. There was a statistically significant difference between the groups (p = 0.032). Statistical tests revealed the group aided by Diagnocat had a superior performance in comparison to ground truth.

**Table 5:** Sensitivity and specificity by condition for aided and unaided AI system (Diagnocat).

Condition	Sensitivity		Specificity	
	Aided	Unaided	Aided	Unaided
Artificial crown	0.8337	0.6774	0.9804	0.971
Canals N = 1	0.9544	0.9417	0.9655	0.9515
Canals N = 2	0.793	0.7345	0.9713	0.9632
Canals N = 3	0.828	0.7347	0.9719	0.9666
Canals N = 4	0.7345	0.751	0.9807	0.9719
Canals N = 5	N/A	N/A	0.9998	0.9992
Caries signs	0.6693	0.6634	0.8593	0.7973
Crown defect over 50%	0.9088	0.7887	0.9901	0.9812
Endodontically treated tooth	0.9761	0.9476	0.9912	0.9841
Filling	0.9308	0.7771	0.9515	0.9151
Impaction	0.8523	0.5	0.9993	0.9993
Implant	1.0	0.969	1.0	0.9996
Missed canal	0.8233	0.7701	0.9867	0.946
Missing	0.8964	0.8973	0.9403	0.9364
Overfilling	0.7909	0.6199	0.9926	0.9922
PDL widening along the root	0.746	0.2779	0.9803	0.9731
Periapical lesion	0.8304	0.683	0.9388	0.9465
The periapical lesion, PDL widening	0.5161	0.3202	0.9332	0.9542
The periapical lesion, poorly circumscribed radiolucency	0.4318	0.2045	0.9897	0.981
Periapical lesion, well circumscribed radiolucency	0.7135	0.6048	0.9912	0.9817
Periodontal bone loss	0.7239	0.4501	0.8111	0.8358
Periodontal bone loss, mild	0.4783	0.2329	0.8739	0.8782
Periodontal bone loss, moderate	0.4357	0.2148	0.9248	0.943
Periodontal bone loss, severe	0.3535	0.2727	0.9929	0.9878
Pontic	0.9696	0.9087	0.9988	0.9973
Post and core	0.7877	0.7114	0.9846	0.9715
Roots N = 1	0.9559	0.9635	0.9731	0.9558
Roots N = 2	0.8609	0.8304	0.9728	0.9726
Roots N = 3	0.9376	0.9028	0.9712	0.9785
Roots N = 4	0.1818	0.7045	0.9996	0.9986
Short filling	0.8139	0.6285	0.9846	0.9817
Voids present in the root filling	0.7592	0.4938	0.9826	0.9757

The average time for the aided group was 17.55 min while it took 18.74 min for the unaided respectively. There was a significant difference between the two groups (p = 0.032). Statistical tests revealed that the AI-aided group had a lower evaluation period in comparison with the unaided group (p = 0.032).

## Discussion

The integration of AI into healthcare has dramatically accelerated in the past decade. The use of deep learning advanced almost synchronously in both medical and dental fields^9^. Previous studies in dentistry focused on image-processing algorithms to achieve high-accuracy classification and segmentation in dental radiographs. They used mathematical morphology, active contour models, level-set methods, Fourier descriptors, textures, Bayesian techniques, linear models, or binary support vector machines^27,41^. However, image components are usually obtained manually using these image-enhancement algorithms. The deep learning method used in this AI system (Diagnocat) yielded fairer outcomes by automatically obtaining image features. The objects detected in an image are classified into a pretrained network without preliminary diagnostics, as a result of processes such as filtering and subdivision. With its direct problem-solving ability, deep learning is used extensively in medicine. Deep learning methods using CNNs are a cornerstone of medical image analysis^42^. Such methods have been preferred in AI studies in dental radiology as well. Tooth detection, identification, and numeration are the first diagnostic steps in dental radiography. Image-processing algorithms have been developed with classification and segmentation in dental radiographs using mathematical morphology, active contour, or level-set methods. A previous study used Bayesian classification for generating an automated dental identification system to classify and identify teeth in bitewing radiographs^25^. Similarly, another study recommended a tooth classification and numbering system to efficiently segment, classify, and number teeth using an image enhancement technique in bitewing radiographs^43^. Tooth detection and numbering have been researched intensively during the last few decades mainly using threshold and region-based techniques. CNN as a popular deep learning method has been used to detect and number teeth as well. It was also emphasized that localization of teeth is important for dental image applications, similarly to our results^44^. In this paper, the authors suggested an original teeth localization technique for periapical radiographs using oriented tooth detection using a CNN. The results of this study showed that the proposed method is effective to localize teeth successfully.

Similar CBCT studies were performed and reported in the literature. One of them considered automatic teeth classifying system using 7 types of axial slice CBCT images using a CNN^37^. The authors concluded that a 7-tooth type classification system can be used efficiently for automatic dental charting^37^. Another study also described a CNN model modified with AlexNet architecture for tooth detection in panoramic radiographs^38^. This study defined mouth gap detection that showed the possible placement of teeth for preprocessing steps. It was concluded that this model could be efficiently used for the detection of teeth. These findings are in line with our study. Another study^45^ used the sea mask region-based CNN method with transfer learning strategies while another^46^ used a fully deep learning mask region-based convolutional neural network (R-CNN) method implemented through a fine-tuning process for automated tooth segmentation. This technique showed high performance for automatic teeth segmentation on panoramic radiographs. Similarly, one more study also used the state-of-the-art Faster R-CNN model of tooth detection and numbering^47^. A recently published paper proposed a deep learning CNN model with a VGG16 network structure for the teeth detection and classification of periapical radiographs^27^. The CNN method is similar to our study, which can also interfere with deep learning methods and can be used for both training and transfer learning.

In 2018 we published our AI algorithm (later called Diagnocat)^48^ that presented coarse-to-fine volumetric segmentation of teeth in CBCT images which were efficient for handling large volumetric images for tooth segmentation. Diagnocat's approach in diagnosing is based on a deep convolutional neural network using a U-net-like architecture^49^. The problem formulation in the study in terms of machine learning tasks was semantic segmentation, including segmenting background and periapical pathology. Specificity and sensitivity metrics were used to evaluate diagnostic performance and to measure the localization capabilities of our model, binary voxel-wise intersection over union (IoU) of the ground truth mask and prediction were used. In the current study, we tested the diagnostic performance of this AI system compared to the experienced dentomaxillofacial examiners. Then, we compared the diagnostic performance and required diagnostic time within aided and unaided groups in a real-time clinical environment. To the best of our knowledge, there is no study to assess the real-time clinical performance of CBCT imaging and diagnosis to demonstrate the clinical safety and effectiveness of its use. In the first study, ground truth set-up results showed the sensitivity and specificity values for human examiners in between 0.9318 and 0.9438 while the value for this AI system was 0.9239. The inter-observer variations among human examiners for the aforementioned significant variables can be interpreted as the diagnosis of these variables does not agree among human observers. For instance, caries diagnosis is a clinical judgment regarding the presence of the disease. Caries lesion detection is the identification of caries signs, clinically or radiographically. However, dentists in general practice should keep in mind that even a clinically sound tooth may contain extensive dentinal caries. Bitewing radiography has a low sensitivity for the detection of early proximal lesions that extends only into the enamel. This leads to the assumption that early lesions (extending in the outer enamel histologically) will usually remain undetected^50,51^. Moreover, caries detection is not a primary indication for a CBCT scan. The SEDENTEXCT Panel in 2011 concluded that the evidence did not support the clinical use of CBCT for caries detection and diagnosis. Nonetheless, CBCT examinations performed for other purposes should be carefully examined for caries lesions shown fortuitously when performing a clinical evaluation^52^. Thus, these fine anatomical details together with caries and periodontal bone loss evaluations are prone to different diagnoses amongst human observers. However, it should be stated that radiographic evaluations performed with the Diagnocat are found to be more compatible and accurate in detecting dental caries in CBCT images.

For the second study, it was shown that the AI-aided group had superior performance compared to the unaided group. The group with AI support had 0.85 averaged by condition sensitivity value and 0.97 specificity value as opposed to 0.77 and 0.96 for the unaided group, correspondingly. AI-system reduced the average time needed to assess one CBCT by 1.19 min (6.78%) due to automatic preparation of dental charts and electronic dental records together with automated pathology detection. Dentists, as well as radiologists, can use AI systems as an ancillary tool to enhance the accuracy of diagnosis, aid treatment planning, and predict treatment outcomes. The use of decision support systems as a second opinion can improve the accuracy of diagnosis within a shorter time frame. Regarding clinical documentation, reporting is generally time-consuming for radiologists. Besides, the report generation task is usually completed by the end of the workflow, which may lead to errors from preceding steps. Moreover, a lack of standardization has led to a variation in documentation among radiologists. Combined with appropriate training, AI can ensure that radiologists produce highly valuable data, improving the efficiency and accuracy of documentation. By reducing inefficiencies, radiologists can have a broader and deeper impact on patient care.

In this study, the results showed that the specificity and sensitivity of the evaluation process were improved in the AI-aided group and the required diagnosis time was reduced. Since examiners' evaluations were taken as the ground truth, this comparison is biased in favor of human examiners. Moreover, the difference in overall performance seems to be rather small, which we interpreted as possible proof of Diagnocat standalone capability.

Nonetheless, the current study has some limitations. The major limitation of this study is its retrospective design. Additionally, the study population was restricted to only three centers with three different CBCT machines. We expect that the classification power of the developed AI system will be enhanced when it is more trained and validated on larger patients data from multiple institutions. Furthermore, this AI system can be directly used in clinical practice to provide additional information for decision-making. Besides, when generating such AI systems, the annotation and segmentation method might take more time and effort. While the ground truth is determined by the hybrid approach in this retrospective study, unfortunately, true diagnosis cannot be made without histopathological evaluation. Also, although inter-observer variability among observers was evaluated in this study, no intra-observer variability was tested due to large database evaluations with various anatomy and pathological aspects. Further studies are needed for every pathology one by one. Another limitation for this study is that no attempt was made for the evaluation of confounding factors such as artifacts (beam hardening, scattering, motion artifacts, etc.), since beam hardening, motion artifacts may affect the diagnosis. As it was stated previously we excluded these conditions from our database.

Within the limitations of the study, we found there was no statistically significant difference between Diagnocat and the experienced dentomaxillofacial radiologists (p > 0.05). The results of sensitivity and specificity values were also similar between this AI system and the examiners.

Given the rapid advances in AI, it seems likely that radiology and pathology images will be examined at some point by a machine. Speech, voice, and text recognition are already aided by computerized systems and software. Patient communication and dictation of clinical notes are only a few examples of everyday computerized tasks, and their diversity will more likely increase. AI systems have the potential to be used in daily routine dental practice. These AI systems will not replace human clinicians on a large scale, but rather will augment the dentists’ efforts to care for patients. However, it should be noted that regulators must approve the widespread adoption of such AI systems for routine dental use.

In conclusion, the null hypothesis of this study was rejected as there is a significant difference between aided and unaided groups and as the proposed AI system (Diagnocat) significantly improved the sensitivity and specificity in regards to diagnosing the dental pathologies in comparison to human observers using CBCT imaging. Our results look promising qualitatively and quantitatively. Diagnocat can have many uses in the real world, ranging from being a decision support system in clinical settings to a helper system for dental practitioners.

## Methods

### Ethics and information governance

Written informed consent was obtained from all patients before CBCT examinations. The research protocol was performed following the principles of the Declaration of Helsinki and was approved by the non-interventional Institutional Review Board (IRB) within the research project of the YDU 82-1147 of Near East University, Faculty of Medicine, Health Sciences Ethics Committee (Nicosia, Cyprus). Deidentification was performed in line with the Information Commissioner's Anonymization: managing data protection risk code of practice (https://ico.org.uk/media/1061/anonymisati code.pdf), and validated by the aforementioned institution. Only deidentified anonymized retrospective data were used for research, without the active involvement of patients.

CBCT images were taken from 3 clinics in DICOM format and anonymized for use in this study. CBCT machines that were used in this study were namely, Ortophos XG unit (Ortophos XG3D; Sirona; Bensheim; Germany), Carestream Health (Carestream Health CS 8100 3D; Kodak; New York; USA), PaX-i3D Smart (PaX-i3D Smart PHT-30LFO0; Vatech; Hwaseong-si; Gyeonggi-do; Korea). The scanners offer multiple fields of view, allowing the dentist to select the optimum scan on a case-by-case basis. Images were obtained using a 4 × 4 field of view (FoV) to 10 × 10 FoV between 0.100 and 0.200 mm^3^ voxel sizes with isotropic voxels.

### Testing the system

The primary goal of this study was to evaluate the ability of this AI system (Diagnocat) to enhance the diagnostic capabilities of the dentist and radiologist. To test this, a few steps had to be taken to prepare the dataset for viewing and analysis. These steps are necessary due to the inherent variability of CBCT datasets coming from CBCT machines as well as the variability in clinical experience on the part of the examiners. Thus, this study has two distinct parts. The first was preparing the dataset for evaluation and the second was evaluating the usefulness of the system for enhancing diagnostic capabilities.

#### Part (A): Preparing the dataset for evaluation

##### Image processing

Due to the high variety of CBCT scanning devices and different calibration settings, CBCT images need to be normalized for both manual and automatic diagnostics. This is usually done with the help of such DICOM properties as window level and window width extracted from scan metadata. Unfortunately, the radiodensity of bone and tissue scans from the same scanning device manufacturer differs when the extracted window is applied. The difference is significantly higher when corresponding windows are applied to images from different devices. Normalization of radiodensity measured in Hounsfield Units (HU) is a basic requirement for computer-aided diagnostics^53,54^ that ensures the similar performance of the system on images from different scanners and imaging protocols. Our normalization process comprises clipping outlier HU values and basic standardization:

1. HU values below − 1000 (air radiodensity) are clipped.
2. HU values below 5th and above 95th percentiles of an image are clipped.
3. HU values are standardized by subtracting the mean value and dividing the difference by the standard deviation.

The last step may vary depending on the task. When there is no need to preserve the difference between a dense bone (2000 HU) and metal (3000 HU) radiodensities, HU values above 2000 can be clipped, and resulting values can be rescaled to [0, 1] range.

##### Localization datasets

To obtain precise segmentation results for training purposes, dental and radiology specialists used ITK-SNAP software (http://itksnap.org, USA)^55^ that allows users to navigate 3D images in three planes. Once being annotated, each segmentation mask was automatically examined to eliminate human factors, e.g. misalignment of a tooth volume and a resulting mask.

The localization modules have different datasets sharing the properties of data origin and variety in scanner origin, FoV, voxel size, and artifacts presence. CBCT scans were sourced from several clinic chains in Moscow, Russia. Each dataset includes a different number of CBCT scans depending on the data counts required to achieve desired module performance, the annotation difficulty, module-specific requirements of data variety, and module region of interest (e.g. 1 CBCT image can be a single input for tooth localization module and a sample of 32 inputs for periodontitis module).

- *ROI (region of interest) localization module*. The dataset consists of 562 CBCT scans with segmented teeth and jaws. The scans are equally distributed among 19 scanner models of 12 scanner manufacturers.
- *Tooth localization and numeration module.* The dataset consists of 684 CBCT scans with segmented and numerated teeth. The scans are equally distributed among 24 scanner models of 15 scanner manufacturers.
- *Periodontitis module.* The dataset consists of 99 CBCT scans with the precisely segmented alveolar bone area and 120 CBCT scans with precisely segmented enamel area of teeth. The scans are equally distributed among 11 scanner models of 8 scanner manufacturers. Each side of a tooth (mesial, distal, oral, and vestibular) has a group of three periodontium landmark points: a point of cementoenamel junction, a point of bone attachment, and a point of bone peak within 2 mm tooth vicinity. The dataset with segmented enamel area is used to obtain the first point, while the dataset with segmented alveolar bone is used to obtain two latter points.
- *Caries localization module.* The dataset consists of 4398 tooth volumes with a context area. The class labels are background (no pathology), caries sign, metallic artifact, and non-contrast filling. One instance can have multiple conditions. A lead radiologist (KO) additionally validated the dataset.
- *Periapical lesion localization module.* The dataset consists of 2800 tooth volumes with a context area. The class labels are background (no pathology), PDL widening, poorly circumscribed radiolucency, well-circumscribed radiolucency, and radiopacity. One instance can have multiple conditions. A lead radiologist (KO) additionally validated the dataset.

##### Classification (descriptor) datasets

Descriptor, the main diagnostic module, is a complex model that, besides accurate data collection, requires several iterations of dataset formation and annotation regulations. We provided a detailed description of the annotation process and insights on managing class imbalance and high model uncertainty.

- *Annotation protocol.* Every radiologist was provided with an instruction describing annotation including a list of required pathologies, access to the internal web-based application that provided a data collection form, and an option to download study DICOM for standalone viewing. Additionally, every radiologist reviewed and described 3 sample CBCTs containing all target pathologies, which were then reviewed by the study supervisor, a highly experienced oral and maxillofacial radiologist (KO). Then, the study supervisor provided feedback to the radiologist. Each radiologist independently studied a CBCT image in a clinical viewer software and noted the presence or absence of each condition for each tooth in the target list. Examiners used the CBCT viewer software Planmeca Romexis (Romexis 5.1; Helsinki; Finland) which they were already comfortable with and used in their clinical practice. Radiologists were required to answer either “applicable”, or “not applicable” for every condition in Table 5.
- *Initial annotation.* During the first stage of the annotation process, a group of experienced radiologists annotated a large set of images following the annotation protocol. Images were randomly sampled, filtered by the study coordinator according to the inclusion and exclusion criteria, and then passed to radiologists. Before the main annotation process, annotators were trained and evaluated by the study coordinator:
  - Participant studied annotation instruction and protocol.
  - Participant annotated a small set of exemplary images, the study coordinator evaluated the results and provided feedback to the participant.

During this stage, each sample (distinct patient-tooth) received 1 diagnostic vote for every condition in consideration.

- *Test set separation.* Following the completion of the first stage of annotation, a test was separated from the annotated data pool and excluded from all following development activities. Test images were sampled in a way to have at least N positives and N negatives for every condition. The choice of N, equal to 300, was motivated by the available number of positive samples for rare conditions. The sampling procedure was as follows.
  1. Randomly sample a condition.
  2. If the test set contained less than N positives of the condition, sample a random positive example from the data pool and allocate it to the test set. Additionally, allocate all other samples from the same image.
  3. Repeat until the test set contains at least N positives and N negatives for each condition.

Each sampled example contains annotation for all target conditions, so the resulting test set contains more than N positives and N negatives for the majority of conditions. Additionally, the test set contains a different number of positives and negatives for each condition, typically, negatives outnumbering the positives (class imbalance). This influenced our decision to choose the AUPRC metric for evaluation as it is robust to significant class imbalance. Moreover, to balance the tendency to predict positive and negative classes before testing, model outputs underwent probabilistic calibration^56^. To obtain well-calibrated probabilities and achieve good classification performance at the threshold 0.5 (which was used as the model’s single operating point), model softmax scores were rescaled by optimizing p = aq ÷ (aq + (1 − a) × (1 − q)) where $a$ is a model uncalibrated score, $p$ is the resulting calibrated probability, and $q$ is a parameter. Parameter $q$ was selected by optimizing the F1 score over the development set.

- *Test set additional annotation.* An additional vote from a second radiologist (SA) was obtained for each tooth condition (sample). Then, for the sample where the first two radiologists disagreed, another vote from the third radiologist was obtained (DT). Ground truth was decided by the majority vote (2-vote agreement).
- *Model development dataset.* A set for model development purposes formed from the remaining annotated data pool (i.e. not included in the test set) was split into training and validation subsets as it was fit for the task. As the majority of examples in the train set had only 1 vote, it was expected that some labels would be incorrect. However, deep learning is known to have some level of robustness against noisy training labels, and we hypothesized that the models would be able to learn the correct labels and achieve satisfactory scores. Additionally, the partially trained model could be used to find and correct the erroneous votes by measuring disagreement between votes and model predictions. In the course of this project, this hypothesis was confirmed. While samples with 1 vote were widely used in the train set, model validation was performed using standard 2-vote agreement protocol.
- *Rare case mining.* Following the separation of the train set, a series of models was trained. Then, the best model was used to enrich the train set by mining rare cases and finding potentially erroneous votes in the train set. Initially rare conditions did not contain enough positive examples in the train set. To rectify this, the following mining procedure was implemented:
  1. Define a set of rare condition lists where additional data is required.
  2. Perform inference of the best model available at a time on studies from the non-annotated data pool.
  3. Calculate information entropy for every condition in the rare condition list.
  4. Sample teeth with high information entropy.
  5. Run images containing sampled teeth through the annotation process.

Information entropy is defined as

$$ S = - P_{i} \log P_{i} $$

where *P*_*i*_ is the probability of the ith outcome of a set of all possible outcomes. For binary tasks, such as our formulation, *i* iterates over “present” and “not present”. Information entropy is highest when probabilities of “present” and “not present” are equal to 0.5. Intuitively, information entropy is a measure of uncertainty in the probability distribution. High uncertainty on an example excluded from the training and validation set means that the training process is likely to improve if the example is annotated and added to the training set.

- *Incorrect vote mining and rectification.* Since we collected only 1 vote for a large number of images allocated to the train set, some of these votes were submitted incorrectly. To rectify this, we implemented the following procedure:
  1. Perform *K*-fold inference on all images in the train set using the best model available at a time. *K*-fold inference procedure:
    - Split train images into *K* disjoint sets
    - Pick a subset *i*
    - Train model on all subsets except *i*
    - Perform inference onset *i* and record resulting scores
    - Repeat for every *i* in *K*
  2. Calculate radiologist-model disagreement.
  3. Sample from cases where radiologist-model disagreement was high.
  4. Collect additional votes for sampled cases using the annotation process.
- *Dataset statistics.* We evaluated the performance of our models on a dataset with 705,017 samples consisting of 28,745 teeth and 25 conditions across 1135 CBCT scans. The scans are distributed among 31 scanner models of 17 scanner manufacturers.

**Figure 1:**
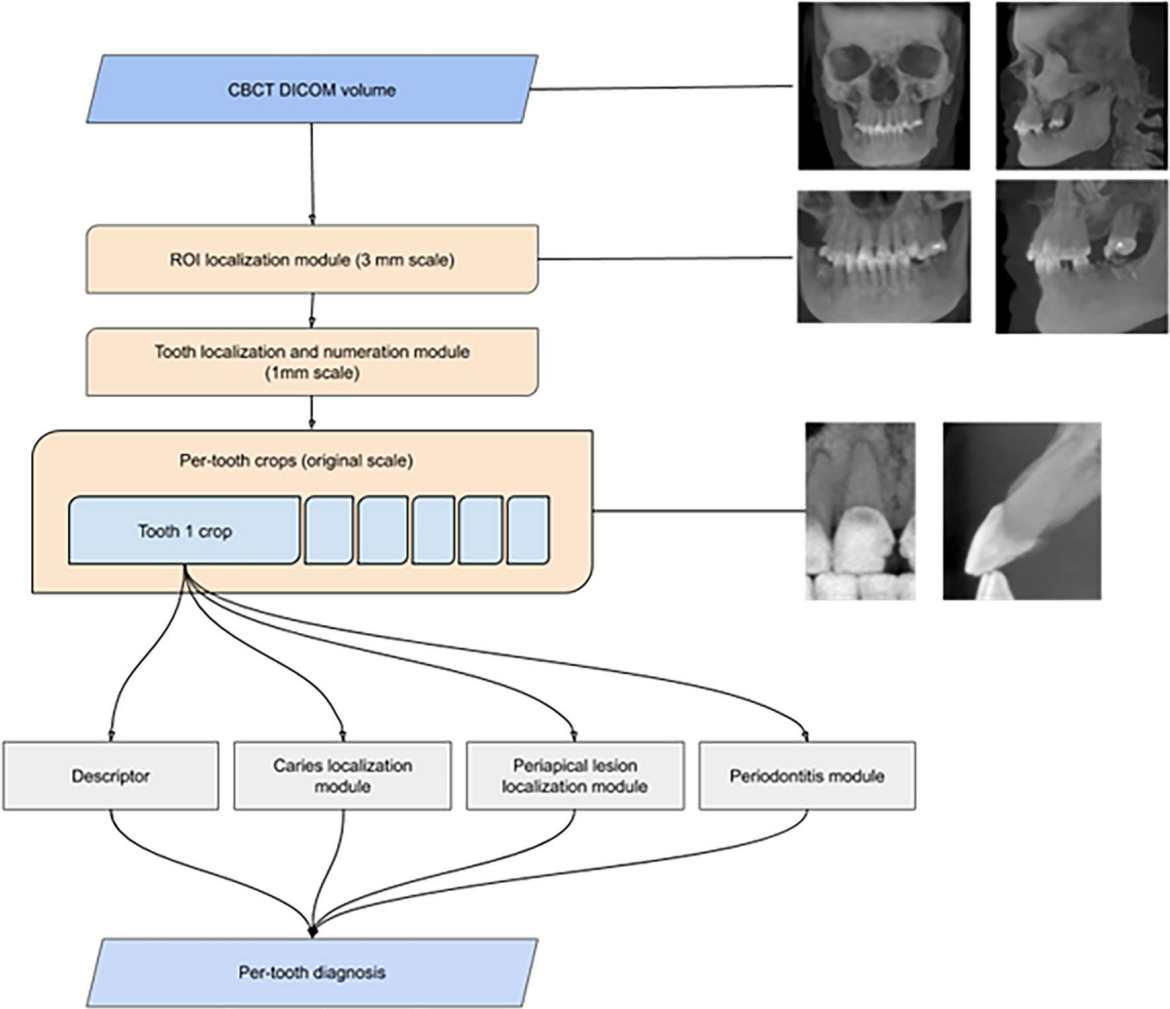
Flow diagram of CBCT processing pipeline and workflow of the AI system.

The flow of the system pipeline is shown in Fig. 1. Each step analyzes data in progressively higher spatial resolution, from a coarse voxel size of 3 mm^3^ at the initial stage of ROI localization to a fine voxel size up to 0.15 mm^3^ for final per-tooth diagnosis. This multi-step pathway was required due to the large memory size of CBCT images at the original resolution.

The first step is the ROI localization module (Fig. 1). Reduction of FoV to ROI sufficient for analysis of dental diseases allows completion of the diagnostics without any information loss. The ROI localizer identifies specific regions of jaws and teeth with some extended context and excludes other anatomical regions. The localization module is based on the volumetric modification of U-Net architecture^57^ performing 3-class semantic segmentation: background, teeth, and jaw bone. To fit large FoV volumes, the module operates at 3 mm^3^ per voxel resolution.

The cropped image is further passed to the tooth localization and numeration module (Fig. 1) that plays a crucial role in the diagnostic pipeline. Tooth localization allows further analysis of different conditions inside and around a tooth, while tooth numeration helps with determining number-specific attributes and inter-tooth relations. The localization and numeration module is implemented as a volumetric U-Net architecture network performing semantic segmentation on 54 classes (the background, 52 possible teeth, and an additional class for supernumerary teeth). It operates at 1 mm^3^ per voxel resolution.

At the next step, each localized tooth area is extended with some context and passed to Descriptor (Fig. 1) that defines the probabilities of a tooth being affected by a set of conditions (Table 1). The descriptor is a principal classification module and is implemented as an ensemble of a ResNeXt^58^ architecture with integrated squeeze-and-excitation blocks^59^ and a DenseNet^60^ architecture performing multiple binary classifications on 25 classes.

**Figure 2:**
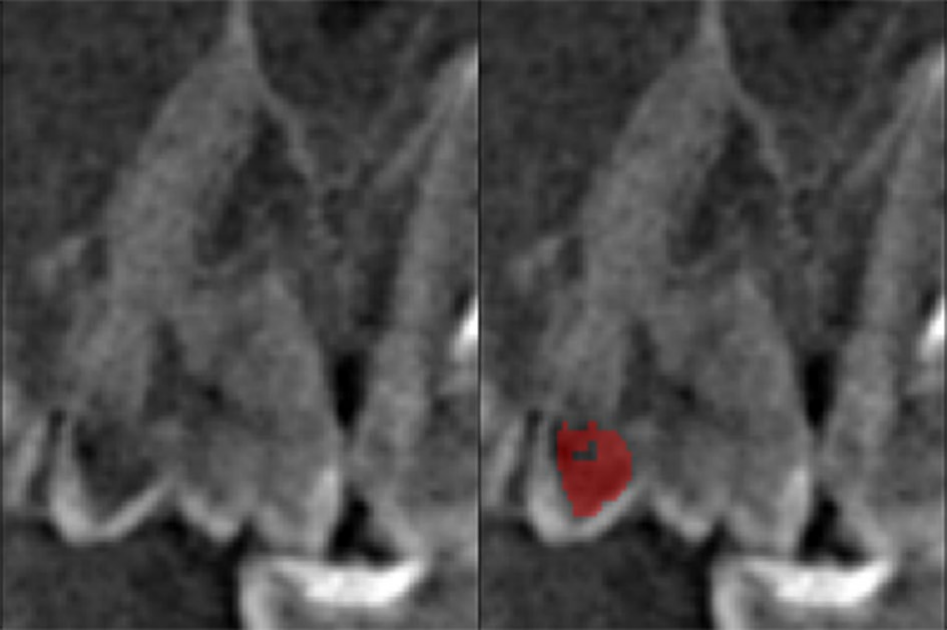
Caries lesion localization mask at the sagittal slice of tooth 17.

**Figure 3:**
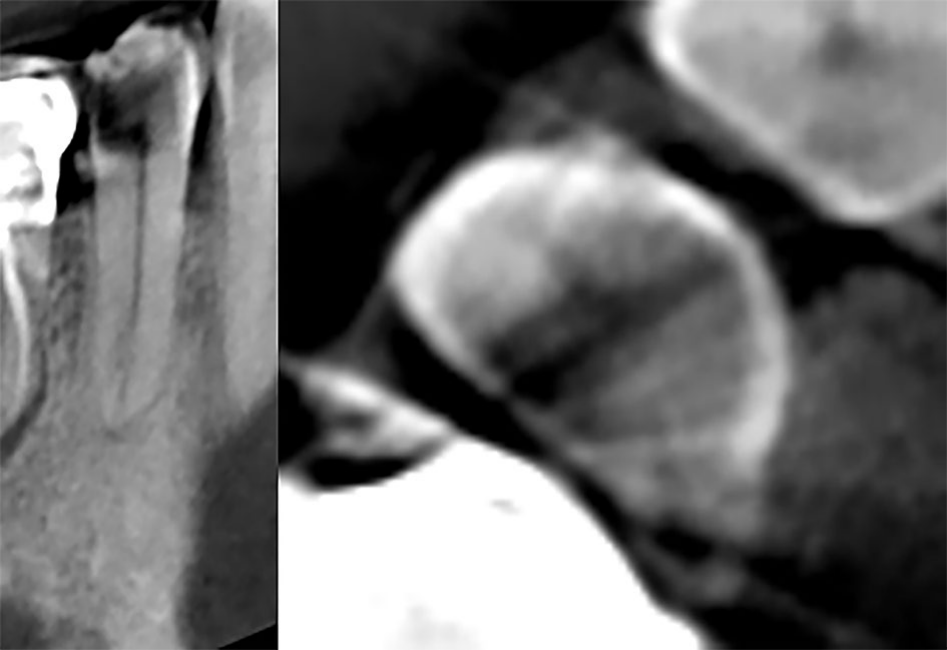
Caries lesion at mesiodistal and axial slices of tooth 45. No caries predicted by caries localization module. The identification of caries was overlooked due to metallic artifacts which is an example of incorrect classification by the AI system.

**Figure 4:**
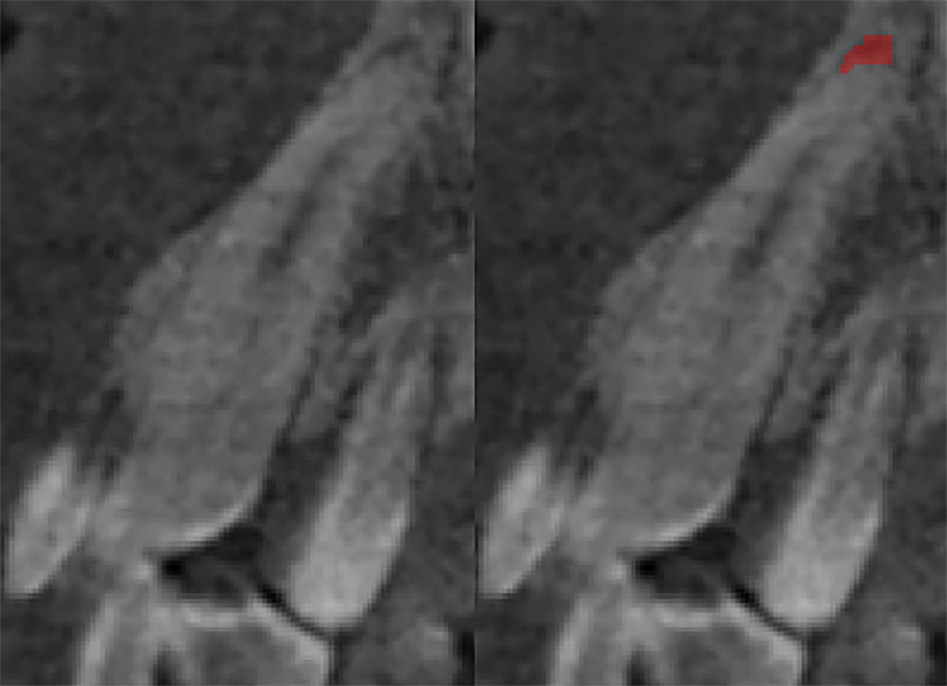
Periapical lesion localization mask at tooth 13.

**Figure 5:**
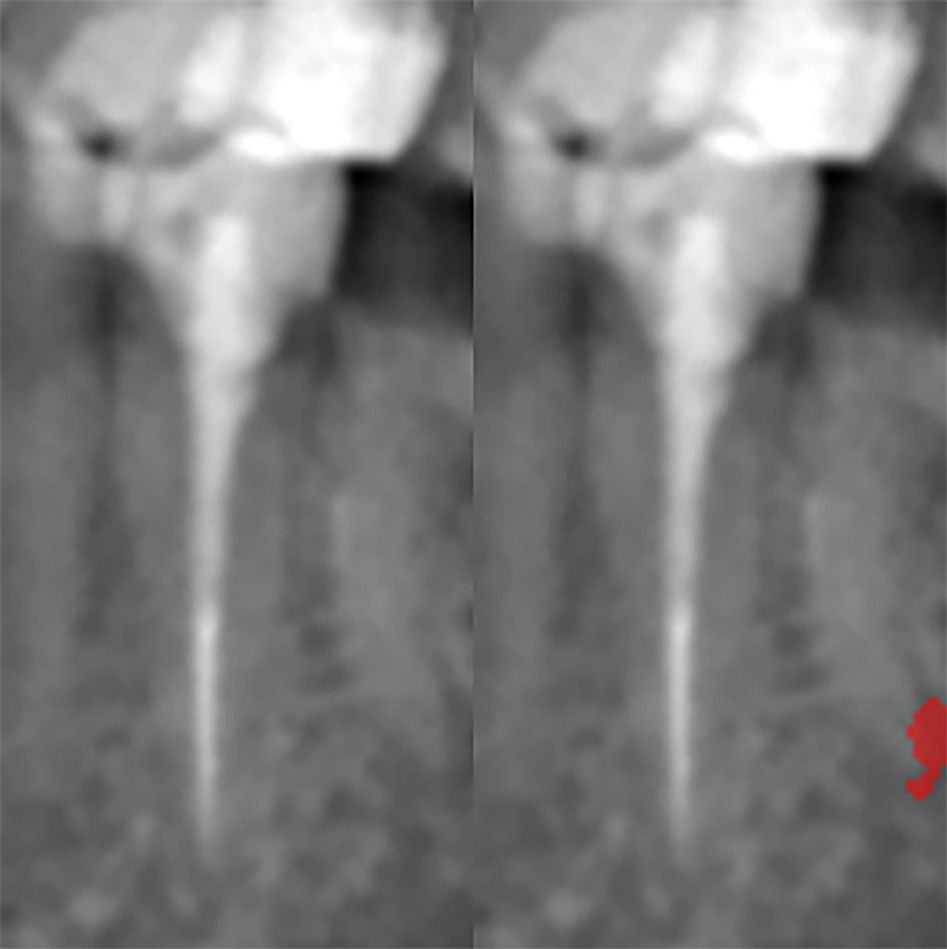
Incorrect periapical lesion localization mask at tooth 45. The lesion was predicted at the adjacent tooth 46.

Three modules for auxiliary classification purposes further examine each tooth volume. (1) The periodontitis module detects and evaluates alveolar bone loss in close vicinity to a tooth. It allows the classification of 3 bone loss types of different severity by calculating distances between pairs of periodontium landmarks segmented by a separate landmark localizer. (2) The caries localization module defines signs of caries probability using segmentation of carious lesions found inside a tooth area (Figs. 2, 3). (3) The periapical lesion localization module detects periapical lesion presence and allows the classification of 4 lesion types found around a tooth (Figs. 4, 5). The embedded localizers of three classification modules are implemented as volumetric U-Net architecture networks performing semantic segmentation.

#### Part (B) Evaluating the ability of the AI system (Diagnocat) to enhance the diagnostic capabilities of the dentist and radiologist

##### Evaluating diagnostic capabilities of the Diagnocat AI system

The primary endpoint was to test the end-to-end performance of this AI system, measuring tooth localization, numeration, and diagnostic sub-modules as a single system. It allowed us to estimate the overall safety and performance of the proposed system.

The Diagnocat AI software was used to obtain a binary condition prediction made on 3D CBCT scans using its predefined operating point (checkpoints of the trained models), which was then compared to ground truth to calculate sensitivity (proportion of correctly defined conditions) and specificity (proportion of correctly defined teeth not having conditions).

The secondary endpoint was to evaluate examiners' performance and compare it to the AI results. Although examiners were tested on the data that was beforehand annotated by each of them the results showed the comparable diagnostic quality of Diagnocat and the examiners. For the performance evaluation, a set of 300 CBCT maxillofacial images in DICOM format was sourced consecutively from three clinics (100 images from each site). CBCT machines that were used in this part were also from the same machines, namely, 3 different CBCT scanners were included in the study to test the reliability of AI systems in different CBCT scanners and setup. CBCT machines that were used in this study were namely, Orthophos XG unit (Orthophos XG3D; Sirona; Bensheim; Germany), Carestream Health (Carestream Health CS 8100 3D; Kodak; New York; USA), PaX-i3D Smart (PaX-i3D Smart PHT-30LFO0; Vatech; Hwaseong-si; Gyeonggi-do; Korea). The scanners offer multiple fields of view, allowing the dentist to select the optimum scan on a case-by-case basis. Images were obtained using a 4 × 4 FOV to 10 × 10 FOV between 0.100 and 0.200 mm^3^ voxel sizes with isotropic voxels.

All images are anonymized by replacing “PatientName” with an empty string and truncating “PatientBirthDate” to the first day of the nearest year. Subsequently, images were screened against the inclusion and exclusion criteria.

The inclusion criteria were:

- a patient with the ability to consent to participate in the project
- a patient of 21 years or older
- anonymized CBCT image of the maxillofacial region, and
- Both model and manufacturer of imaging devices are not present in the training dataset of the system (allows testing generalizability to new imaging devices).

The exclusion criteria were:

- images containing significant motion artifacts (as judged by the radiologist coordinating the study)
- images containing severe artifacts such as streak artifacts, beam hardening (low and medium artifact remover was applied using device-specific software when available for standardization of the images) and;
- images of patients with cleft lip and palate, trauma, bone lesions, and severe bone erosions.

A scientific coordinator (KO—an internationally recognized dentomaxillofacial radiologist with at least 18 years of experience) then reviewed the final set of images and 10 images were rejected due to significant motion artifacts. To establish the ground truth, examiners were recruited from experienced dentomaxillofacial radiologists. In total, the data was evaluated by four of them with a mean of 10 years of professional experience.

Each examiner was responsible for the annotation of CBCT anatomy on their own. Moreover, the examiners were unaware of the conditions of the patients. Each examiner was then trained by the study coordinator to annotate 3D CBCT scans and fill the provided form correctly. After the study coordinator evaluated the examiners and approved them as sufficiently trained, the study proceeded to actual data collection. Each radiologist received a random, non-overlapping portion of the dataset via electronic means (shared folder). They evaluated the cases in their clinical environment and filled the spreadsheet, then saved them to separate shared folders. The examiners could not access each other's forms. After they evaluated cumulatively and annotated the full CBCT dataset, the second round of annotation started, where the examiners were assigned a different random subset of the dataset. After the second round was finished, the third commenced. At the end of the third round, the scientific coordinator collected the examination from 3 radiologists for every sample. Evaluations took place between December 2019 and April 2020. Data were extracted on an individual and group comparison level. To establish the true values of conditions, a consensus process was performed, where the ground truth was taken as a majority (at least 2 of 3 votes) per each case, tooth, and condition. The whole process was then reviewed again by the study coordinator for last adjustments and to establish final ground truth evaluations of each patient and teeth as well as for each condition. Inference of the Diagnocat system was performed once for the full dataset: an engineer performed inference using the production version of the system.

##### Evaluating the clinical performance

After evaluation of the diagnostic capability of the Diagnocat AI system, the next step is to evaluate the clinical performance of the system which can be achieved by comparing the accuracy of the diagnosis and time required for the reading for aided and unaided cases. An independent clinical staff recorded the time for evaluations with a timer. Evaluation duration was compared between aided and unaided to determine if the addition of Diagnocat suggestions changes the time required to review the case. It was estimated that approximately eight weeks were required to conduct this study. This was addressed at each stage, from recruitment to analysis: recruitment and consent—1 week, training and randomization—1 week, investigation—1 week, washout—at least 1 month, investigation—1 week, and analysis—2 weeks. The washout period was at least 1 month. This was to minimize memory bias and confounding factors. Crossover design reduced confounding factors as well. To identify the number of required examiners a power analysis was performed^61^. To hold the study at least 20 examiners were required in total. Thus, 24 dentists were enrolled in the study as the examiners and divided into two groups at a 1:1 ratio: (1) Group 1 examined the CBCTs with AI system-aided; (2) Group 2 examined the CBCTs unaided. Both groups during the study worked with the CBCT viewer software Planmeca Romexis (Romexis 5.1; Helsinki; Finland). The aided group also used the proposed system via a web-based interface. The aided group examined both the visual features of a tooth and the suggested classification and then decided to either keep the suggested classification or revise and change it. The confidence interval is 0.80, and 5% was used for error. Enrolled examiners were qualified general dental practitioners of various experiences with no defined specialty interest. Following inclusion and exclusion criteria were applied:

- Inclusion criteria
  1. Qualified dentist—General Dental Practitioner
  2. At least 5 years of experience in dentistry
  3. Ability to interpret dental CBCT
  4. Access to CBCT software at the workplace
- Exclusion criteria
  1. Unable to commit to the study
  2. Employees of Diagnocat—and their relatives were excluded from participation

The scientific advisor for CBCT scan reading conducted training sessions for examiners for one hour including the use of the Diagnocat AI system. 10 training CBCT scans were used for training and practice purposes; those encompassed the full spectrum of required diagnostics. A list of all possible diagnoses was given as well to ensure that the scope of diagnosis was calibrated and participants were aware of that (Tables 2, 3, 4). There was also remote support available to guide through the training process. For this study, the overall dataset contained 40 CBCT images, including 30 study images and 10 images for examiners' training. These scans were sampled randomly from the dataset of the standalone performance test. 30 study images were sequentially numbered after randomization was performed. Thus each participant had a different sequence of clinical cases. Each CBCT scan required all 32 teeth to be diagnosed with none, one or more pathologies. Thus, 32 units in each CBCT, multiplied by the number of pathologies identified in each unit, with a total number of 30 CBCT scans per group. In this way, 960 (30 × 32 = 960) diagnostic activities were carried out in each investigation by each participant. The crossover nature of this study ensured that this was performed twice by each participant. Table 5 shows the conditions that were asked to diagnose by the examiners. Once investigations were completed, the raw data from forms filled by the unaided group and Diagnocat was transformed into the same format using automated scripts written before the study and then sent to an independent blinded assessor. This same assessor for both groups analyzed the data and compared it with ground truth (same as in the standalone Diagnocat performance test). Raw data were compared to ground truth electronically. Scoring was performed via electronic means and data stored securely. Once this was completed, groups were decoded and results compared. The output of this process was a set of condition detections, where for every tooth and condition model outputs a probability distribution along with predicted tooth number. This evaluation was calculated only once during this study and was not used for system training purposes. Moreover, during this process engineers did not have access to the examiners' votes data. To score the results, an independent expert was provided with both ground truth and Diagnocat inference data. The outside expert was responsible for running data analysis and producing performance measurements for primary and secondary endpoints.

### Examiner reliability and statistical analysis

Statistical analyses were carried out using the SPSS 21.0 software (SPSS, Chicago, IL, USA). Intra- and inter-examiner validation measures were conducted. The inter-observer reliability was determined by the intraclass correlation coefficient (ICC) and the coefficient of variation (CV) [CV = (standard deviation ÷ mean) × 100%]. Values for the ICC range from 0 to 1. ICC values greater than 0.75 show good reliability, and the low CV demonstrates the precision error as an indicator for reproducibility^62^. The student's t-test was performed for statistical analysis of variables (p < 0.05).

The differences between examiners in aided vs unaided groups as well as the time for evaluating the CBCT images were also calculated. Data were assessed for normality using the Kolmogorov–Smirnov test. For group comparisons, Spearman correlation analyses and Mann Whitney-u test were used. A value of p < 0.05 was considered statistically significant.

## Supplementary Information

Supplementary Information.
