# Author Correction: Clinically applicable artificial intelligence system for dental diagnosis with CBCT

**DOI:** 10.1038/s41598-021-01678-5

**Published:** 2021-11-09

**Authors:** Matvey Ezhov, Maxim Gusarev, Maria Golitsyna, Julian M. Yates, Evgeny Kushnerev, Dania Tamimi, Secil Aksoy, Eugene Shumilov, Alex Sanders, Kaan Orhan

**Affiliations:** 1 Diagnocat Inc, San Francisco, CA USA; 2 Division of Dentistry, School of Medical Sciences, The University of Manchester, Manchester, UK; 3 Private Practice, Orlando, FL USA; 4 Department of DentoMaxillofacial Radiology, Faculty of Dentistry, Near East University, Nicosia, Cyprus; 5 Department of DentoMaxillofacial Radiology, Faculty of Dentistry, Ankara University, 06500 Ankara, Turkey; 6 Medical Design Application and Research Center (MEDITAM), Ankara University, Ankara, Turkey

Correction to: *Scientific Reports* https://doi.org/10.1038/s41598-021-94093-9, published online 22 July 2021

In the original version of this Article, the Competing Interests statement was incomplete. The text,

“Financial support was received by Diagnocat Co. Ltd., San Francisco CA. Matvey Ezhov, Maxim Gusarev, Maria Golitsyna, Eugene Shumilov, and Alex Sanders are employees of Diagnocat Co. Ltd. Julian M Yates, Evgeny Kushnerev, Dania Tamimi, Secil Aksoy, and Kaan Orhan declare no potential competing interests.”

now reads as follows:

“Financial support was received by Diagnocat Co. Ltd., San Francisco CA. Matvey Ezhov, Maxim Gusarev, Maria Golitsyna, Eugene Shumilov, and Alex Sanders are employees of Diagnocat Co. Ltd. Kaan Orhan is a scientific research advisor for the Diagnocat Co. Ltd., San Francisco CA. Julian M Yates, Evgeny Kushnerev, Dania Tamimi, Secil Aksoy have no potential competing interests."

The original Article has been corrected.

