# Supplementary material for: Clinically applicable artificial intelligence system for dental diagnosis with CBCT
Source: Sci Rep. 2021 Jul 22;11:15006. doi: 10.1038/s41598-021-94093-9 (PMC8298426; doi:10.1038/s41598-021-94093-9)
Supplement: Supplementary file 1 — Supplementary Information. [file 41598_2021_94093_MOESM1_ESM.pdf]

Clinically Applicable AI System for Dental Diagnosis with CBCT

Matvey Ezhov[1], Maxim Gusarev[1], Maria Golitsyna[1], Julian M Yates[2], Evgeny Kushnerev[3], Dania Tamimi[4], Secil Aksoy[5], Eugene Shumilov[1], Alex Sanders[1], Kaan Orhan[6,7]

Supplements

Supplement 1 – Examiner forms

	A	B	C	D	E	F	G	H	I	J	K	L	M	N	O	P	Q	R	S	T	U	V	W	X	Y	Z	AA	AB	AC	AD	AE	AF	AG
1	Write case number here	18	17	16	15	14	13	12	11	21	22	23	24	25	26	27	28	38	37	36	35	34	33	32	31	41	42	43	44	45	46	47	48
2	Artificial crown																																
3	Canals N=1																																
4	Canals N=2																																
5	Canals N=3																																
6	Canals N=4																																
7	Canals N=5																																
8	Caries signs																																
9	Crown defect over 50pct																																
10	Endodontically treated tooth																																
11	Filling																																
12	Impaction																																
13	Implant																																
14	Missed canal																																
15	Missing																																
16	Overfilling																																
17	PDL widening along root																																
18	Periapical lesion																																
19	Periapical lesion, PDL widening																																
20	Periapical lesion, poorly circumscribed radiolucency																																
21	Periapical lesion, radiopacity																																
22	Periapical lesion, well circumscribed radiolucency																																
23	Periodontal bone loss																																
24	Periodontal bone loss, mild																																
25	Periodontal bone loss, moderate																																
26	Periodontal bone loss, severe																																
27	Pontic																																
28	Post and core																																
29	Roots N=1																																
30	Roots N=2																																
31	Roots N=3																																
32	Roots N=4																																
33	Short filling																																
34	Voids present in the root filling																																
35																																	

A screenshot of unfilled Excel form that examiners used to record the diagnoses. Participants would first record the case number in A1 cell, then start reviewing the case tooth-by-tooth, and put any marks in the cells corresponding to tooth-condition diagnosis they consider to be present. All unfilled cells were considered as negative diagnosis, all filled cells as positive.

Participants underwent comprehensive training and examination to ensure they are able to comfortably use and fill the forms.

Supplement 2 – Sensitivity and Specificity values for examiners and the device

condition	participant	tn	tp	fn	fp	sensitivity	specificity
Artificial crown	device	8847	442	21	33	0.9546 (0.9359 - 0.9681)	0.9963 (0.9951 - 0.9972)
	DMFR-1	6298	336	10	12	0.9711 (0.9522 - 0.9826)	0.9981 (0.997 - 0.9988)
	DMFR-2	7105	342	14	27	0.9607 (0.94 - 0.9744)	0.9962 (0.9948 - 0.9972)
	DMFR-3	7385	376	27	20	0.933 (0.9095 - 0.9507)	0.9973 (0.9961 - 0.9981)
	DMFR-4	5924	302	9	5	0.9711 (0.9509 - 0.9831)	0.9992 (0.9983 - 0.9996)
Canals N=1	device	4907	4206	58	172	0.9864 (0.9832 - 0.989)	0.9661 (0.9617 - 0.9701)
	DMFR-1	3562	3017	35	42	0.9885 (0.9849 - 0.9913)	0.9883 (0.985 - 0.9909)
	DMFR-2	4025	3330	104	29	0.9697 (0.9645 - 0.9742)	0.9928 (0.9903 - 0.9947)
	DMFR-3	4185	3491	85	47	0.9762 (0.9717 - 0.9801)	0.9889 (0.9859 - 0.9912)
	DMFR-4	3378	2776	34	52	0.9879 (0.984 - 0.9909)	0.9848 (0.981 - 0.9879)
Canals N=2	device	8194	845	244	60	0.7759 (0.7545 - 0.796)	0.9927 (0.991 - 0.9941)
	DMFR-1	5864	697	66	29	0.9135 (0.8953 - 0.9288)	0.9951 (0.9933 - 0.9964)
	DMFR-2	6450	870	35	133	0.9613 (0.9493 - 0.9706)	0.9798 (0.9767 - 0.9825)
	DMFR-3	6800	871	57	80	0.9386 (0.9243 - 0.9503)	0.9884 (0.986 - 0.9903)
	DMFR-4	5500	632	61	47	0.912 (0.8926 - 0.9281)	0.9915 (0.9892 - 0.9933)
Canals N=3	device	7656	1341	66	280	0.9531 (0.9429 - 0.9615)	0.9647 (0.9611 - 0.968)
	DMFR-1	5604	930	69	53	0.9309 (0.9165 - 0.943)	0.9906 (0.9883 - 0.9925)
	DMFR-2	6274	1042	102	70	0.9108 (0.896 - 0.9237)	0.989 (0.9866 - 0.9909)
	DMFR-3	6548	1114	81	65	0.9322 (0.9193 - 0.9432)	0.9902 (0.988 - 0.992)
	DMFR-4	5260	861	56	63	0.9389 (0.9246 - 0.9507)	0.9882 (0.9855 - 0.9904)
Canals N=4	device	8933	240	127	43	0.654 (0.6121 - 0.6935)	0.9952 (0.9939 - 0.9963)
	DMFR-1	6298	283	20	55	0.934 (0.9065 - 0.9538)	0.9913 (0.9892 - 0.9931)
	DMFR-2	7145	255	50	38	0.8361 (0.7983 - 0.868)	0.9947 (0.9931 - 0.9959)
	DMFR-3	7429	288	28	63	0.9114 (0.8815 - 0.9343)	0.9916 (0.9897 - 0.9932)
	DMFR-4	6049	147	41	3	0.7819 (0.7286 - 0.8273)	0.9995 (0.9988 - 0.9998)
Canals N=5	device	9341	0	0	2		0.9998 (0.9994 - 0.9999)
	DMFR-1	6656	0	0	0		1.0 (0.9996 - 1.0)
	DMFR-2	7488	0	0	0		1.0 (0.9996 - 1.0)
	DMFR-3	7808	0	0	0		1.0 (0.9997 - 1.0)
	DMFR-4	6240	0	0	0		1.0 (0.9996 - 1.0)
Caries signs	device	8940	263	98	42	0.7285 (0.6884 - 0.7652)	0.9953 (0.994 - 0.9964)
	DMFR-1	6376	222	20	38	0.9174 (0.8834 - 0.9421)	0.9941 (0.9923 - 0.9955)
	DMFR-2	7066	250	66	106	0.7911 (0.7511 - 0.8262)	0.9852 (0.9827 - 0.9874)
	DMFR-3	7254	277	19	258	0.9358 (0.9082 - 0.9555)	0.9657 (0.962 - 0.9689)
	DMFR-4	5958	201	36	45	0.8481 (0.8058 - 0.8825)	0.9925 (0.9904 - 0.9941)
Crown defect over 50pct	device	9241	69	10	23	0.8734 (0.7993 - 0.9228)	0.9975 (0.9965 - 0.9982)
	DMFR-1	6593	54	2	7	0.9643 (0.8977 - 0.9881)	0.9989 (0.998 - 0.9994)
	DMFR-2	7418	51	10	9	0.8361 (0.7442 - 0.8994)	0.9988 (0.9979 - 0.9993)
	DMFR-3	7721	63	4	20	0.9403 (0.8735 - 0.9729)	0.9974 (0.9963 - 0.9982)
	DMFR-4	6178	49	10	3	0.8305 (0.7361 - 0.8959)	0.9995 (0.9988 - 0.9998)
Endodontically treated tooth	device	8348	925	31	39	0.9676 (0.9568 - 0.9758)	0.9953 (0.994 - 0.9964)
	DMFR-1	5919	720	11	6	0.985 (0.9756 - 0.9908)	0.999 (0.998 - 0.9995)
	DMFR-2	6678	783	8	19	0.9899 (0.9821 - 0.9943)	0.9972 (0.9959 - 0.998)
	DMFR-3	7023	750	16	19	0.9791 (0.9688 - 0.9861)	0.9973 (0.9961 - 0.9981)

condition	participant	tn	tp	fn	fp	sensitivity	specificity
	DMFR-4	5621	594	20	5	0.9674 (0.9534 - 0.9773)	0.9991 (0.9982 - 0.9996)
Filling	device	6991	2232	64	56	0.9721 (0.9659 - 0.9772)	0.9921 (0.9901 - 0.9936)
	DMFR-1	4915	1694	26	21	0.9849 (0.9792 - 0.989)	0.9957 (0.9939 - 0.997)
	DMFR-2	5586	1766	54	82	0.9703 (0.9631 - 0.9762)	0.9855 (0.9827 - 0.9879)
	DMFR-3	5839	1884	46	39	0.9762 (0.9698 - 0.9812)	0.9934 (0.9914 - 0.9949)
	DMFR-4	4762	1425	37	16	0.9747 (0.967 - 0.9806)	0.9967 (0.995 - 0.9978)
Impaction	device	9199	127	12	5	0.9137 (0.8662 - 0.9454)	0.9995 (0.9989 - 0.9997)
	DMFR-1	6556	93	4	3	0.9588 (0.9113 - 0.9814)	0.9995 (0.9989 - 0.9998)
	DMFR-2	7363	114	5	6	0.958 (0.9162 - 0.9794)	0.9992 (0.9984 - 0.9996)
	DMFR-3	7682	115	6	5	0.9504 (0.907 - 0.9741)	0.9993 (0.9987 - 0.9997)
	DMFR-4	6155	78	4	3	0.9512 (0.8957 - 0.9779)	0.9995 (0.9988 - 0.9998)
Implant	device	9230	107	3	3	0.9727 (0.9337 - 0.989)	0.9997 (0.9992 - 0.9999)
	DMFR-1	6568	85	1	2	0.9884 (0.9496 - 0.9974)	0.9997 (0.9991 - 0.9999)
	DMFR-2	7414	72	1	1	0.9863 (0.9409 - 0.9969)	0.9999 (0.9994 - 1.0)
	DMFR-3	7712	91	2	3	0.9785 (0.9371 - 0.9929)	0.9996 (0.999 - 0.9998)
	DMFR-4	6159	74	4	3	0.9487 (0.8906 - 0.9768)	0.9995 (0.9988 - 0.9998)
Missed canal	device	9201	79	39	24	0.6695 (0.5952 - 0.7362)	0.9974 (0.9964 - 0.9981)
	DMFR-1	6522	90	12	32	0.8824 (0.8197 - 0.9252)	0.9951 (0.9935 - 0.9963)
	DMFR-2	7376	77	25	10	0.7549 (0.6789 - 0.8178)	0.9986 (0.9977 - 0.9992)
	DMFR-3	7695	83	11	19	0.883 (0.8174 - 0.9271)	0.9975 (0.9964 - 0.9983)
	DMFR-4	6164	48	22	6	0.6857 (0.589 - 0.7686)	0.999 (0.9981 - 0.9995)
Missing	device	7287	1567	28	461	0.9824 (0.9762 - 0.9871)	0.9405 (0.9359 - 0.9448)
	DMFR-1	5445	966	151	94	0.8648 (0.8471 - 0.8808)	0.983 (0.9799 - 0.9857)
	DMFR-2	6202	1178	35	73	0.9711 (0.9621 - 0.9781)	0.9884 (0.9859 - 0.9904)
	DMFR-3	6391	1244	60	113	0.954 (0.9435 - 0.9626)	0.9826 (0.9798 - 0.9851)
	DMFR-4	4933	1122	52	133	0.9557 (0.9447 - 0.9646)	0.9737 (0.9698 - 0.9772)
Overfilling	device	9129	148	41	25	0.7831 (0.7299 - 0.8282)	0.9973 (0.9962 - 0.998)
	DMFR-1	6505	124	17	10	0.8794 (0.827 - 0.9175)	0.9985 (0.9974 - 0.9991)
	DMFR-2	7313	153	5	17	0.9684 (0.9364 - 0.9845)	0.9977 (0.9966 - 0.9984)
	DMFR-3	7630	158	8	12	0.9518 (0.9165 - 0.9726)	0.9984 (0.9975 - 0.999)
	DMFR-4	6121	94	16	9	0.8545 (0.7908 - 0.9013)	0.9985 (0.9975 - 0.9991)
PDL widening along root	device	8937	248	34	124	0.8794 (0.8439 - 0.9078)	0.9863 (0.9842 - 0.9882)
	DMFR-1	6395	189	25	47	0.8832 (0.8422 - 0.9146)	0.9927 (0.9907 - 0.9943)
	DMFR-2	7304	88	87	9	0.5029 (0.4411 - 0.5645)	0.9988 (0.9979 - 0.9993)
	DMFR-3	7443	253	9	103	0.9656 (0.9419 - 0.9799)	0.9864 (0.984 - 0.9884)
	DMFR-4	6004	185	19	32	0.9069 (0.8679 - 0.9352)	0.9947 (0.9929 - 0.996)
Periapical lesion	device	8468	700	135	40	0.8383 (0.8163 - 0.8582)	0.9953 (0.9939 - 0.9964)
	DMFR-1	6022	540	45	49	0.9231 (0.9029 - 0.9393)	0.9919 (0.9898 - 0.9936)
	DMFR-2	6768	606	46	68	0.9294 (0.9111 - 0.9442)	0.9901 (0.9879 - 0.9918)
	DMFR-3	6966	659	47	136	0.9334 (0.9163 - 0.9473)	0.9809 (0.978 - 0.9833)
	DMFR-4	5618	523	70	29	0.882 (0.8584 - 0.902)	0.9949 (0.993 - 0.9962)
Periapical lesion, PDL widening	device	8740	327	104	172	0.7587 (0.7233 - 0.7909)	0.9807 (0.9782 - 0.983)
	DMFR-1	6307	218	59	72	0.787 (0.7439 - 0.8246)	0.9887 (0.9863 - 0.9907)
	DMFR-2	7104	217	106	61	0.6718 (0.6276 - 0.7132)	0.9915 (0.9895 - 0.9931)
	DMFR-3	7214	353	25	216	0.9339 (0.9096 - 0.952)	0.9709 (0.9675 - 0.974)
	DMFR-4	5860	253	73	54	0.7761 (0.7359 - 0.8117)	0.9909 (0.9886 - 0.9927)

condition	participant	tn	tp	fn	fp	sensitivity	specificity
Periapical lesion, poorly circumscribed radiolucency	device	9243	32	14	54	0.6957 (0.5758 - 0.7938)	0.9942 (0.9927 - 0.9954)
	DMFR-1	6599	30	3	24	0.9091 (0.7931 - 0.9631)	0.9964 (0.9949 - 0.9974)
	DMFR-2	7436	20	14	18	0.5882 (0.448 - 0.7155)	0.9976 (0.9965 - 0.9984)
	DMFR-3	7748	32	8	20	0.8 (0.6785 - 0.8835)	0.9974 (0.9963 - 0.9982)
	DMFR-4	6190	26	5	19	0.8387 (0.7038 - 0.9192)	0.9969 (0.9956 - 0.9979)
Periapical lesion, radiopacity	device	9339	0	4	0	0.0 (0.0 - 0.4035)	1.0 (0.9997 - 1.0)
	DMFR-1	6644	4	0	8	1.0 (0.5965 - 1.0)	0.9988 (0.9979 - 0.9993)
	DMFR-2	7478	1	3	6	0.25 (0.0579 - 0.6438)	0.9992 (0.9985 - 0.9996)
	DMFR-3	7800	2	1	5	0.6667 (0.2535 - 0.9217)	0.9994 (0.9987 - 0.9997)
	DMFR-4	6238	1	0	1	1.0 (0.2699 - 1.0)	0.9998 (0.9993 - 1.0)
Periapical lesion, well circumscribed radiolucency	device	9057	191	71	24	0.729 (0.6817 - 0.7717)	0.9974 (0.9963 - 0.9981)
	DMFR-1	6415	197	13	31	0.9381 (0.9048 - 0.9603)	0.9952 (0.9936 - 0.9964)
	DMFR-2	7212	201	13	62	0.9393 (0.9065 - 0.961)	0.9915 (0.9895 - 0.9931)
	DMFR-3	7579	184	29	16	0.8638 (0.8206 - 0.898)	0.9979 (0.9968 - 0.9986)
	DMFR-4	6060	140	30	10	0.8235 (0.7705 - 0.8664)	0.9984 (0.9972 - 0.999)
Periodontal bone loss	device	7664	1338	72	269	0.9489 (0.9384 - 0.9577)	0.9661 (0.9626 - 0.9693)
	DMFR-1	5464	906	137	149	0.8686 (0.8505 - 0.8849)	0.9735 (0.9697 - 0.9768)
	DMFR-2	6270	963	75	180	0.9277 (0.9134 - 0.9399)	0.9721 (0.9685 - 0.9753)
	DMFR-3	6451	797	332	228	0.7059 (0.6832 - 0.7277)	0.9659 (0.962 - 0.9693)
	DMFR-4	5052	907	135	146	0.8704 (0.8524 - 0.8866)	0.9719 (0.9679 - 0.9754)
Periodontal bone loss, mild	device	8356	714	52	221	0.9321 (0.9156 - 0.9456)	0.9742 (0.9713 - 0.9769)
	DMFR-1	5964	447	98	147	0.8202 (0.7916 - 0.8456)	0.9759 (0.9725 - 0.979)
	DMFR-2	6757	537	64	130	0.8935 (0.871 - 0.9125)	0.9811 (0.9782 - 0.9836)
	DMFR-3	7008	418	213	169	0.6624 (0.6308 - 0.6927)	0.9765 (0.9733 - 0.9792)
	DMFR-4	5614	426	105	95	0.8023 (0.7723 - 0.8291)	0.9834 (0.9803 - 0.9859)
Periodontal bone loss, moderate	device	8774	410	40	119	0.9111 (0.8865 - 0.9308)	0.9866 (0.9845 - 0.9885)
	DMFR-1	6258	269	71	58	0.7912 (0.7527 - 0.8251)	0.9908 (0.9886 - 0.9926)
	DMFR-2	7101	291	33	63	0.8981 (0.8671 - 0.9226)	0.9912 (0.9892 - 0.9928)
	DMFR-3	7381	244	102	81	0.7052 (0.6634 - 0.7438)	0.9891 (0.987 - 0.9909)
	DMFR-4	5826	306	46	62	0.8693 (0.8369 - 0.8961)	0.9895 (0.987 - 0.9914)
Periodontal bone loss, severe	device	9124	169	13	37	0.9286 (0.8905 - 0.9541)	0.996 (0.9947 - 0.9969)
	DMFR-1	6492	124	20	20	0.8611 (0.807 - 0.9019)	0.9969 (0.9956 - 0.9979)
	DMFR-2	7340	100	17	31	0.8547 (0.7931 - 0.9003)	0.9958 (0.9944 - 0.9969)
	DMFR-3	7646	99	35	28	0.7388 (0.6721 - 0.7961)	0.9964 (0.995 - 0.9973)
	DMFR-4	6044	125	26	45	0.8278 (0.7716 - 0.8725)	0.9926 (0.9906 - 0.9942)
Pontic	device	9252	81	8	2	0.9101 (0.8474 - 0.9486)	0.9998 (0.9993 - 0.9999)
	DMFR-1	6592	55	6	3	0.9016 (0.8209 - 0.9483)	0.9995 (0.9989 - 0.9998)
	DMFR-2	7419	63	1	5	0.9844 (0.933 - 0.9965)	0.9993 (0.9986 - 0.9997)
	DMFR-3	7733	72	3	0	0.96 (0.9041 - 0.9839)	1.0 (0.9997 - 1.0)
	DMFR-4	6168	68	2	2	0.9714 (0.9173 - 0.9905)	0.9997 (0.999 - 0.9999)
Post and core	device	9092	171	57	23	0.75 (0.7001 - 0.7941)	0.9975 (0.9965 - 0.9982)
	DMFR-1	6440	133	48	35	0.7348 (0.6777 - 0.785)	0.9946 (0.9929 - 0.9959)
	DMFR-2	7252	179	9	48	0.9521 (0.9195 - 0.9719)	0.9934 (0.9917 - 0.9948)
	DMFR-3	7609	144	35	20	0.8045 (0.7513 - 0.8485)	0.9974 (0.9962 - 0.9982)
	DMFR-4	6089	123	25	3	0.8311 (0.7746 - 0.8757)	0.9995 (0.9988 - 0.9998)
Roots N=1	device	4400	4694	199	50	0.9593 (0.9544 - 0.9637)	0.9888 (0.9859 - 0.9911)

condition	participant	tn	tp	fn	fp	sensitivity	specificity
	DMFR-1	3129	3439	43	45	0.9877 (0.9842 - 0.9904)	0.9858 (0.9819 - 0.9889)
	DMFR-2	3472	3930	37	49	0.9907 (0.9878 - 0.9929)	0.9861 (0.9824 - 0.989)
	DMFR-3	3607	4075	54	72	0.9869 (0.9837 - 0.9895)	0.9804 (0.9763 - 0.9838)
	DMFR-4	3033	3116	74	17	0.9768 (0.972 - 0.9808)	0.9944 (0.9917 - 0.9962)
Roots N=2	device	7787	1266	113	177	0.9181 (0.9051 - 0.9294)	0.9778 (0.9749 - 0.9803)
	DMFR-1	5623	953	32	48	0.9675 (0.9569 - 0.9756)	0.9915 (0.9893 - 0.9933)
	DMFR-2	6309	1079	50	50	0.9557 (0.9445 - 0.9647)	0.9921 (0.9901 - 0.9938)
	DMFR-3	6597	1097	55	59	0.9523 (0.9408 - 0.9616)	0.9911 (0.989 - 0.9928)
	DMFR-4	5286	855	45	54	0.95 (0.9366 - 0.9607)	0.9899 (0.9874 - 0.9919)
Roots N=3	device	8328	803	30	182	0.964 (0.9518 - 0.9732)	0.9786 (0.9759 - 0.981)
	DMFR-1	6012	589	30	25	0.9515 (0.9353 - 0.9639)	0.9959 (0.9943 - 0.997)
	DMFR-2	6795	635	35	23	0.9478 (0.9317 - 0.9602)	0.9966 (0.9953 - 0.9976)
	DMFR-3	7065	686	29	28	0.9594 (0.9455 - 0.9699)	0.9961 (0.9946 - 0.9971)
	DMFR-4	5688	499	15	38	0.9708 (0.9559 - 0.9808)	0.9934 (0.9914 - 0.9949)
Roots N=4	device	9334	0	9	0	0.0 (0.0 - 0.2311)	1.0 (0.9997 - 1.0)
	DMFR-1	6647	9	0	0	1.0 (0.7689 - 1.0)	1.0 (0.9996 - 1.0)
	DMFR-2	7478	7	1	2	0.875 (0.5889 - 0.9716)	0.9997 (0.9992 - 0.9999)
	DMFR-3	7802	4	2	0	0.6667 (0.347 - 0.8827)	1.0 (0.9997 - 1.0)
	DMFR-4	6236	4	0	0	1.0 (0.5965 - 1.0)	1.0 (0.9996 - 1.0)
Short filling	device	8873	289	125	56	0.6981 (0.6598 - 0.7338)	0.9937 (0.9922 - 0.995)
	DMFR-1	6291	315	25	25	0.9265 (0.8997 - 0.9465)	0.996 (0.9945 - 0.9971)
	DMFR-2	7053	319	28	88	0.9193 (0.8919 - 0.9402)	0.9877 (0.9853 - 0.9896)
	DMFR-3	7428	294	36	50	0.8909 (0.8594 - 0.916)	0.9933 (0.9916 - 0.9947)
	DMFR-4	5984	197	52	7	0.7912 (0.7458 - 0.8303)	0.9988 (0.9978 - 0.9994)
Voids present in the root filling	device	9027	203	74	39	0.7329 (0.687 - 0.7742)	0.9957 (0.9944 - 0.9967)
	DMFR-1	6391	207	24	34	0.8961 (0.8584 - 0.9247)	0.9947 (0.993 - 0.996)
	DMFR-2	7211	191	40	46	0.8268 (0.7822 - 0.8639)	0.9937 (0.9919 - 0.995)
	DMFR-3	7544	187	29	48	0.8657 (0.823 - 0.8994)	0.9937 (0.992 - 0.995)
	DMFR-4	6059	139	33	9	0.8081 (0.7541 - 0.8526)	0.9985 (0.9975 - 0.9991)

Supplement 3 –Table shows the results of aided and unaided groups as percentages for true and false diagnosis.

		Artificial crown / gt					
		True		False		Total	
		n	%	n	%	n	%
Artificial crown	Aided True	1600	80,9	377	19,1	1977	100,0
	Unaided True	1290	69,7	561	30,3	1851	100,0
	Aided False	314	1,6	18829	98,4	19143	100,0
	Unaided False	624	3,2	18645	96,8	19269	100,0
	Total	3828	9,1	38412	90,9	42240	100,0
		Canals N=1 / gt					
		True		False		Total	
		n	%	n	%	n	%
Canals N=1	Aided True	9826	96,4	371	3,6	10197	100,0
	Unaided True	9699	94,9	525	5,1	10224	100,0
	Aided False	472	4,3	10451	95,7	10923	100,0
	Unaided False	599	5,5	10297	94,5	10896	100,0
	Total	20596	48,8	21644	51,2	42240	100,0
		Canals N=2 / gt					
		True		False		Total	
		n	%	n	%	n	%
Canals N=2	Aided True	2192	80,1	544	19,9	2736	100,0
	Unaided True	2016	74,8	678	25,2	2694	100,0
	Aided False	569	3,1	17815	96,9	18384	100,0
	Unaided False	745	4,0	17681	96,0	18426	100,0
	Total	5522	13,1	36718	86,9	42240	100,0
		n	%	n	%	n	%
Canals N=3	Aided True	2728	84,5	502	15,5	3230	100,0
	Unaided True	2421	80,2	598	19,8	3019	100,0
	Aided False	569	3,2	17321	96,8	17890	100,0
	Unaided False	876	4,8	17225	95,2	18101	100,0
	Total	6594	15,6	35646	84,4	42240	100,0

		Canals N=4 / gt					
		True		False		Total	
		n	%	n	%	n	%
Canals N=4	Aided True	717	64,8	389	35,2	1106	100,0
	Unaided True	724	56,2	564	43,8	1288	100,0
	Aided False	257	1,3	19757	98,7	20014	100,0
	Unaided False	250	1,3	19582	98,7	19832	100,0
	Total	1948	4,6	40292	95,4	42240	100,0

		Canals N=5 / gt					
		True		False		Total	
		n	%	n	%	n	%
Canals N=5	Aided True	0	0,0	4	100,0	4	100,0
	Unaided True	0	0,0	18	100,0	18	100,0
	Aided False	0	0,0	21116	100,0	21116	100,0
	Unaided False	0	0,0	21102	100,0	21102	100,0
	Total	0	0,0	42240	100,0	42240	100,0

		Caries signs / gt					
		True		False		Total	
		n	%	n	%	n	%
Caries signs	Aided True	750	21,1	2803	78,9	3553	100,0
	Unaided True	741	15,4	4065	84,6	4806	100,0
	Aided False	369	2,1	17198	97,9	17567	100,0
	Unaided False	378	2,3	15936	97,7	16314	100,0
	Total	2238	5,3	40002	94,7	42240	100,0

		Crown defect over 50pct / gt					
		True		False		Total	
		n	%	n	%	n	%
Crown defect over 50pct	Aided True	519	72,0	202	28,0	721	100,0
	Unaided True	451	53,8	388	46,2	839	100,0
	Aided False	54	,3	20345	99,7	20399	100,0
	Unaided False	122	,6	20159	99,4	20281	100,0
	Total	1146	2,7	41094	97,3	42240	100,0

		Endodontically treated tooth / gt					
		True		False		Total	
		n	%	n	%	n	%

Endodontically treated tooth	Aided True	3790	96,2	151	3,8	3941	100,0
	Unaided True	3681	92,9	282	7,1	3963	100,0
	Aided False	98	,6	17081	99,4	17179	100,0
	Unaided False	207	1,2	16950	98,8	17157	100,0
	Total	7776	18,4	34464	81,6	42240	100,0

		Filling / gt					
		True		False		Total	
		n	%	n	%	n	%
Filling	Aided True	6049	89,5	707	10,5	6756	100,0
	Unaided True	5060	80,2	1251	19,8	6311	100,0
	Aided False	458	3,2	13906	96,8	14364	100,0
	Unaided False	1447	9,8	13362	90,2	14809	100,0
	Total	13014	30,8	29226	69,2	42240	100,0

		Impaction / gt					
		True		False		Total	
		n	%	n	%	n	%
Impaction	Aided True	88	83,8	17	16,2	105	100,0
	Unaided True	44	74,6	15	25,4	59	100,0
	Aided False	13	,1	21002	99,9	21015	100,0
	Unaided False	57	,3	21004	99,7	21061	100,0
	Total	202	,5	42038	99,5	42240	100,0

		Implant / gt					
		True		False		Total	
		n	%	n	%	n	%
Implant	Aided True	330	99,7	1	,3	331	100,0
	Unaided True	313	97,2	9	2,8	322	100,0
	Aided False	0	0,0	20789	100,0	20789	100,0
	Unaided False	17	,1	20781	99,9	20798	100,0
	Total	660	1,6	41580	98,4	42240	100,0

		Missed canal / gt					
		True		False		Total	
		n	%	n	%	n	%
Missed canal	Aided True	452	62,3	274	37,7	726	100,0

	Unaided True	423	27,6	1108	72,4	1531	100,0
	Aided False	98	,5	20296	99,5	20394	100,0
	Unaided False	127	,6	19462	99,4	19589	100,0
	Total	1100	2,6	41140	97,4	42240	100,0
		Missing / gt					
		True		False		Total	
		n	%	n	%	n	%
Missing / aided	Aided True	2039	64,5	1121	35,5	3160	100,0
	Unaided True	2041	63,0	1200	37,0	3241	100,0
	Aided False	240	1,3	17720	98,7	17960	100,0
	Unaided False	237	1,3	17641	98,7	17878	100,0
	12,0	1	100,0	0	0,0	1	100,0
	Total	4558	10,8	37682	89,2	42240	100,0
		Overfilling / gt					
		True		False		Total	
		n	%	n	%	n	%
Overfilling	Aided True	598	79,4	155	20,6	753	100,0
	Unaided True	465	74,2	162	25,8	627	100,0
	Aided False	157	,8	20210	99,2	20367	100,0
	Unaided False	290	1,4	20203	98,6	20493	100,0
	Total	1510	3,6	40730	96,4	42240	100,0
		PDL widening along root / gt					
		True		False		Total	
		n	%	n	%	n	%
PDL widening along root	Aided True	509	55,8	403	44,2	912	100,0
	Unaided True	190	25,6	551	74,4	741	100,0
	Aided False	174	,9	20034	99,1	20208	100,0
	Unaided False	493	2,4	19886	97,6	20379	100,0
	Total	1366	3,2	40874	96,8	42240	100,0
		Periapical lesion / gt					
		True		False		Total	
		n	%	n	%	n	%
Periapical lesion	Aided True	2278	67,2	1111	32,8	3389	100,0
	Unaided True	1874	65,2	999	34,8	2873	100,0

	Aided False	467	2,6	17264	97,4	17731	100,0
	Unaided False	871	4,8	17376	95,2	18247	100,0
	Total	5490	13,0	36750	87,0	42240	100,0

		Periapical lesion, PDL widening / gt					
		True		False		Total	
		n	%	n	%	n	%
Periapical lesion, PDL widening	Aided True	740	36,2	1302	63,8	2042	100,0
	Unaided True	461	33,6	913	66,4	1374	100,0
	Aided False	692	3,6	18386	96,4	19078	100,0
	Unaided False	971	4,9	18775	95,1	19746	100,0
	Total	2864	6,8	39376	93,2	42240	100,0

		Periapical lesion, poorly circumscribed radiolucency / gt					
		True		False		Total	
		n	%	n	%	n	%
Periapical lesion, poorly circumscribed radiolucency	Aided True	42	16,2	217	83,8	259	100,0
	Unaided True	19	4,5	399	95,5	418	100,0
	Aided False	50	,2	20811	99,8	20861	100,0
	Unaided False	73	,4	20629	99,6	20702	100,0
	Total	184	,4	42056	99,6	42240	100,0

		Periapical lesion, radiopacity / gt					
		True		False		Total	
		n	%	n	%	n	%
Periapical lesion, radiopacity	Aided True	0	0,0	70	100,0	70	100,0
	Unaided True	0	0,0	82	100,0	82	100,0
	Aided False	0	0,0	21050	100,0	21050	100,0
	Unaided False	0	0,0	21006	100,0	21006	100,0
	Total	0	0,0	42208	100,0	42208	100,0

		Periapical lesion, well circumscribed radiolucency / gt					
		True		False		Total	
		n	%	n	%	n	%
Periapical lesion, well circumscribed radiolucency	Aided True	893	83,8	172	16,2	1065	100,0
	Unaided True	756	67,5	364	32,5	1120	100,0
	Aided False	359	1,8	19696	98,2	20055	100,0

	Unaided False	496	2,5	19504	97,5	20000	100,0
	Total	2504	5,9	39736	94,1	42240	100,0

		Periodontal bone loss / gt					
		True		False		Total	
		n	%	n	%	n	%
Periodontal bone loss	Aided True	2535	43,3	3314	56,7	5849	100,0
	Unaided True	1577	35,3	2894	64,7	4471	100,0
	Aided False	973	6,4	14298	93,6	15271	100,0
	Unaided False	1927	11,6	14690	88,4	16617	100,0
	Total	7012	16,6	35196	83,4	42208	100,0

		Periodontal bone loss, mild / gt					
		True		False		Total	
		n	%	n	%	n	%
Periodontal bone loss, mild	Aided True	886	26,8	2425	73,2	3311	100,0
	Unaided True	432	15,5	2347	84,5	2779	100,0
	Aided False	951	5,3	16858	94,7	17809	100,0
	Unaided False	1401	7,7	16908	92,3	18309	100,0
	Total	3670	8,7	38538	91,3	42208	100,0

		Periodontal bone loss, moderate / gt					
		True		False		Total	
		n	%	n	%	n	%
Periodontal bone loss, moderate	Aided True	555	27,2	1486	72,8	2041	100,0
	Unaided True	273	19,3	1143	80,7	1416	100,0
	Aided False	719	3,8	18360	96,2	19079	100,0
	Unaided False	1001	5,1	18671	94,9	19672	100,0
	Total	2548	6,0	39660	94,0	42208	100,0

		Periodontal bone loss, severe / gt					
		True		False		Total	
		n	%	n	%	n	%
Periodontal bone loss, severe	Aided True	71	32,0	151	68,0	222	100,0
	Unaided True	56	17,6	263	82,4	319	100,0
	Aided False	129	,6	20769	99,4	20898	100,0
	Unaided False	144	,7	20657	99,3	20801	100,0
	Total	400	,9	41840	99,1	42240	100,0

		Pontic / gt					
		True		False		Total	
		n	%	n	%	n	%
Pontic	Aided True	256	91,8	23	8,2	279	100,0
	Unaided True	240	78,9	64	21,1	304	100,0
	Aided False	10	,0	20830	100,0	20840	100,0
	Unaided False	26	,1	20790	99,9	20816	100,0
	Total	532	1,3	41707	98,7	42239	100,0
		Post and core / gt					
		True		False		Total	
		n	%	n	%	n	%
Post and core	Aided True	935	75,3	306	24,7	1241	100,0
	Unaided True	843	59,8	566	40,2	1409	100,0
	Aided False	254	1,3	19625	98,7	19879	100,0
	Unaided False	345	1,8	19334	98,2	19679	100,0
	Total	2377	5,6	39831	94,4	42208	100,0
		Roots N=1 / gt					
		True		False		Total	
		n	%	n	%	n	%
Roots N=1	Aided True	11339	97,8	250	2,2	11589	100,0
	Unaided True	11412	96,4	423	3,6	11835	100,0
	Aided False	526	5,5	9004	94,5	9530	100,0
	Unaided False	453	4,9	8832	95,1	9285	100,0
	Total	23730	56,2	18509	43,8	42239	100,0
		Roots N=2 / gt					
		True		False		Total	
		n	%	n	%	n	%
Roots N=2	Aided True	2725	84,9	484	15,1	3209	100,0
	Unaided True	2611	84,1	493	15,9	3104	100,0
	Aided False	434	2,4	17477	97,6	17911	100,0
	Unaided False	548	3,0	17468	97,0	18016	100,0
	Total	6318	15,0	35922	85,0	42240	100,0
		Roots N=3 / gt					
		True		False		Total	
		n	%	n	%	n	%

Roots N=3	Aided True	1879	77,4	548	22,6	2427	100,0
	Unaided True	1804	81,4	413	18,6	2217	100,0
	Aided False	125	,7	18568	99,3	18693	100,0
	Unaided False	200	1,1	18703	98,9	18903	100,0
	Total	4008	9,5	38232	90,5	42240	100,0

		Roots N=4 / gt					
		True		False		Total	
		n	%	n	%	n	%
Roots N=4	Aided True	8	42,1	11	57,9	19	100,0
	Unaided True	31	50,0	31	50,0	62	100,0
	Aided False	36	,2	21065	99,8	21101	100,0
	Unaided False	13	,1	21013	99,9	21026	100,0
	Total	88	,2	42120	99,8	42208	100,0

		Short filling / gt					
		True		False		Total	
		n	%	n	%	n	%
Short filling	Aided True	1642	84,7	296	15,3	1938	100,0
	Unaided True	1267	78,3	352	21,7	1619	100,0
	Aided False	378	2,0	18804	98,0	19182	100,0
	Unaided False	753	3,9	18748	96,1	19501	100,0
	Total	4040	9,6	38200	90,4	42240	100,0

		Voids present in the root filling / gt					
		True		False		Total	
		n	%	n	%	n	%
Voids present in the root filling	Aided True	1239	78,5	339	21,5	1578	100,0
	Unaided True	801	62,9	472	37,1	1273	100,0
	Aided False	389	2,0	19153	98,0	19542	100,0
	Unaided False	827	4,2	19020	95,8	19847	100,0
	Total	3256	7,7	38984	92,3	42240	100,0
